# Foamy Virus Integrase in Development of Viral Vector for Gene Therapy

**DOI:** 10.4014/jmb.2003.03046

**Published:** 2020-07-14

**Authors:** Jinsun Kim, Ga-Eun Lee, Cha-Gyun Shin

**Affiliations:** Department of Systems Biotechnology, Chung-Ang University, Anseong 17546, Republic of Korea

**Keywords:** Foamy virus, integrase, intasome, viral vector

## Abstract

Due to the broad host suitability of viral vectors and their high gene delivery capacity, many researchers are focusing on viral vector-mediated gene therapy. Among the retroviruses, foamy viruses have been considered potential gene therapy vectors because of their non-pathogenicity. To date, the prototype foamy virus is the only retrovirus that has a high-resolution structure of intasomes, nucleoprotein complexes formed by integrase, and viral DNA. The integration of viral DNA into the host chromosome is an essential step for viral vector development. This process is mediated by virally encoded integrase, which catalyzes unique chemical reactions. Additionally, recent studies on foamy virus integrase elucidated the catalytic functions of its three distinct domains and their effect on viral pathogenicity. This review focuses on recent advancements in biochemical, structural, and functional studies of foamy virus integrase for gene therapy vector research.

## Introduction

Since the concept was established in 1972, gene therapy has been studied in concert with the development of molecular biotechnology [1–3]. The first successful trial of gene therapy in humans was performed in 1989 [4]. In that protocol, patients with melanoma were treated with a gene-modified, tumor-infiltrating lymphocyte construct modified by retroviral transduction. There were no side effects from 3 weeks to 2 months during the monitoring period. In 2017, the FDA approved the first gene therapy in the USA. This novel, genetically engineered chimeric antigen receptor T-cell therapy was soon followed by approval of *RPE65* mutation-induced blindness gene therapy. In approximately the last three decades, viral vectors have been used for over 3,000 clinical tasks, from therapy to regenerative medicine, following more than half of them in phase I [5]. Gene therapy has broad prospects as an effective therapeutic strategy for various diseases, including cancer, as well as monogenic, infectious, cardiovascular, and neurological diseases. In terms of types of viruses, adenoviruses, retroviruses, and lentiviruses have been intensively studied, and adeno-associated virus research began recently [5]. However, viral gene therapy vectors pose safety issues, including direct toxicity and stimulation of the immune response [6–8]. Nevertheless, viral vectors have been extensively studied because of their advantages, such as their broad host range, including humans, and high gene transfer rates to host cells.

Among them, the *Retroviridae* family is composed of two virus subfamilies, six genera of *Orthoretrovirinae* and four genera of *Spumaretrovirinae* (commonly called spumaviruses or foamy viruses [FVs]). FVs were first mentioned as a contaminant in primary monkey kidney cultures in 1954 [9]. Since ‘foamy viral agent’ was first isolated in 1955, FV has been found in various mammalians, including humans [10]. FVs are exogenous viruses that induce the specific cytopathic effect (CPE), or ‘foamy’ appearance in host cells [11]. The FV life cycle is different from other retroviruses. First, FV buds from the endoplasmic reticulum instead of the cytoplasmic membrane. This helps form the FV’s unique ‘spike surface’ morphology [11]. Furthermore, the replication of FV is like that of *Hepadnaviridae*, which is another family of virus that has reverse transcriptase-encoding gene. Reverse transcription of FV genome occurs not in the early steps like other retroviruses, but also in the late steps of replication [12]. Although FV-infected cells show characteristic large vacuoles in vitro, there have been no reported serial diseases from FV infection in vivo, so FVs have been considered potential gene therapy vectors [10]. Here, we discuss research achievements with foamy virus vectors (FVVs) in gene therapy and specifically focus on the characteristics of FV integrase (IN) and its prospective functions associated with vector research.

## FVs as Viral Vector Candidates

FVs have a broad range of tissue and cell tropism, and virus infections are generally latent, except in some tissues of the oral cavity. It is highly prevalent in diverse non-primate mammalian animals such as cows, horses, cats, and bats [10]. Interspecies transmission of FVs to humans by exposure to tissue fluids from infected non-human primates has been reported [13, 14]. Unlike other retroviral vector systems, FVV systems do not rely on virus- encoded encapsidation sequences for the transfer of heterologous genetic material, thus enabling the efficient transient expression of FVV [15]. In the last decade, many studies have demonstrated the efficacy of FVV in curing monogenetic diseases of hematopoietic origin in humans, non-human primates, canines, and rodents [16– 20]. In vivo FVV-based gene therapy approaches have been established in the X-linked severe combined immunodeficiency canine model [17, 18]. FVV is also capable of infecting hematopoietic stem progenitor cells [20]. Most recently, it was shown that intramuscular transplantation of FVV-transduced Duchenne muscular dystrophy myoblasts could restore dystrophin for individual small muscles in mice [21].

## Basic Biochemistry of FV IN

Retroviruses are the only viruses that require the integration of genetic information into the host genome as a necessary step for replication [1]. All species of the *Retroviridae* family carry with them an IN, which is a specialized DNA recombination enzyme [22]. Recombinant prototype foamy virus (PFV) IN affords assembly of all key intermediates of retroviral integration, presenting unprecedented experimental approaches to probe interactions between the viral machinery and its cellular partners [23]. Furthermore, PFV IN is a model enzyme for studying the mechanism of retroviral integration. Compared with IN from other retroviruses, PFV IN is highly soluble and more amenable to experimental manipulation [24]. Furthermore, it is sensitive to clinically relevant human immunodeficiency virus (HIV) IN inhibitor, indicating that PFV IN can be used for HIV-1 integrase strand transfer inhibitor (INSTI) screening [25].

**Fig. 1:**
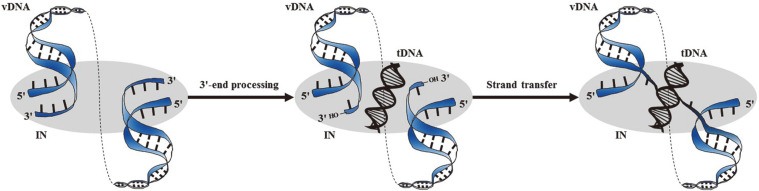
IN catalytic functions and intasome complexes. A multimer of IN (depicted simply by the gray oval) engages the end regions of the linear vDNA molecule (blue), forming a stable synaptic complex. During 3'- end processing, IN hydrolyzes the vDNA ends adjacent to the invariant CA dinucleotides, revealing a set of reactive 3'-hydroxyl groups in the cleaved donor complex. In the strand transfer step, IN employs the 3'-hydroxyl groups as nucleophiles to attack tDNA.

Integration is an essential step for the efficient expression of viral genes by the host transcriptional machinery and, hence, it is also crucial for productive virus replication [22]. Integration occurs within the context of an intasome, which consists of a multimerized IN engaging the two viral DNA (vDNA) ends [26]. The first reaction of retroviral integration is 3'-end processing. In this process, IN removes the terminal dinucleotides following the conserved CA sequence at the 3'-ends of the long terminal repeat (LTR). Secondly, the strand transfer is a phosphoryl transfer reaction in which the 3'-hydroxyl ends of the vDNA donate an electron pair as the nucleophile, resulting in covalent joining between vDNA and target DNA (tDNA) [27, 28]. Disintegration, an additional reaction that occurs in vitro only, is the reverse process of strand transfer in which the inserted vDNA cleaves off from the tDNA [29] (Fig. 1).

Retroviral IN proteins comprise three or four functional domains. The N-terminal domain (NTD), the catalytic core domain (CCD), and the C-terminal domain (CTD) are common to all of the IN proteins [30, 31]. The NTD contains a characteristic zinc-binding HHCC motif, and the CCD harbors a highly conserved acidic triad Asp, Asp35, and Glu, known as the D, D_35_, and E motif, which is essential for the catalysis of 3'-end processing, strand transfer, and disintegration [32]. The INs from the spuma-, epsilon-, and gamma-retroviruses additionally carry the NTD extension domain involved in interactions with vDNA.

The retroviral integration step is accomplished by 3'-end processing and strand transfer steps, which are bimolecular nucleophilic substitution (S_N_^2^) reactions [33–37]. Most retroviral INs remove the terminal dinucleotides (GT in HIV-1, TT in avian sarcoma leukosis virus) from each 3'-end of the vDNA during the 3'-end processing [35, 37]. However, interestingly, FV integration does not require the removal of two terminal nucleotides from its U3 region. In contrast, a terminal dinucleotide (AT) is removed from only its U5 region to provide the subterminal CA sequence for the joining reaction to the host chromosome [36]. In the strand transfer reaction, FV IN employs the free 3'-hydroxyl of the vDNA to attack the tDNA. The subsequent eliminations of the unpaired dinucleotide at each 5'-overhanging end of the vDNA and the filling of the gaps are most likely performed by host enzymes [37]. In terms of structural and mechanistic similarities, retroviral INs belong to a metal-dependent nucleotidyltransferase family. The active sites of these enzymes contain essential acidic residues that participate in coordination with a pair of Mg^2+^ or Mn^2+^ cations [38].

To investigate enzyme catalytic activities and interactions with tDNA in vitro, recombinant IN purification, followed by bacterial expression and integration assays, have been generally used. For enzyme purification from bacterial expression systems, a hexahistidine tag is commonly included in IN expression constructs for initial purification with a nickel resin [24,39-41]. Recombinant IN is commonly fractionated by heparin–Sepharose affinity chromatography with a salt gradient, followed by nickel chromatography for bacterial nuclease-free IN purification. Purified FV INs can perform three types of in vitro enzymatic activities: 3'-end processing, strand transfer, and disintegration [42]. Among them, 3'-end processing and the strand transfer step require specific DNA substrates and intact IN proteins. Furthermore, it has been shown that the IN CCD plays an important role in binding vDNA, and also, in target site selection [43].

Two functional domains engage in metal binding for retroviral integration [38, 39]. One of them is the NTD, which includes a zinc finger motif that binds Zn^2+^. The second domain, the CCD, contains a conserved D, D_35_, and E motif and has been known to bind divalent metal cofactors, such as Mn^2+^ and Mg^2+^. FV IN has low substrate specificity compared with HIV IN. Moreover, the substrate usage of IN is different among FV INs. Specifically, feline foamy virus (FFV) IN has a broader range of substrates than PFV IN. Mn^2+^ or Mg^2+^ metal ions are known as required cofactors of retroviral IN activities. Mg^2+^ is a natural cofactor because of its abundance in vivo, but retroviral IN activities, as well as FV IN in vitro activities, are most efficient in the presence of Mn^2+^ [42, 44]. Some in vitro integration assay studies reported that other transient elements could work as cofactors when FV IN adapts them in the metal-binding sites [44]. According to the results, Co^2+^ and Zn^2+^ ions were found to act in the three kinds of enzymatic activities of FFV IN in the absence of Mn^2+^ ions in vitro, and their inductions of enzymatic reactions were concentration dependent.

FV IN has higher solubility, faster kinetic properties, and a broad range of substrates compared with other retroviruses. These biochemical properties of FV IN facilitate studies of retroviral integration [45].

## Structural Analysis of FV Integration

Numerous biochemical studies have revealed that the active form of retroviral IN is a multimer that engages vDNA and tDNA in the confines of a nucleoprotein complex [46–54]. Bacteriophage Mu-mediated PCR of pre- integration complexes (PICs) extracted from infected cells revealed the protection of several hundred base pairs at the vDNA ends, and the associated complex was termed an “intasome” to distinguish it from the larger PICs [55, 56]. The number of IN monomers per intasome may be four, eight, or possibly higher-order multimers [57]. PFV IN was the first full-length IN protein to be crystallized with vDNAs and tDNAs [58, 59]. Additionally, among the retroviral INs, only PFV IN has yielded diffractable intasome crystals [28, 44].

**Fig. 2:**
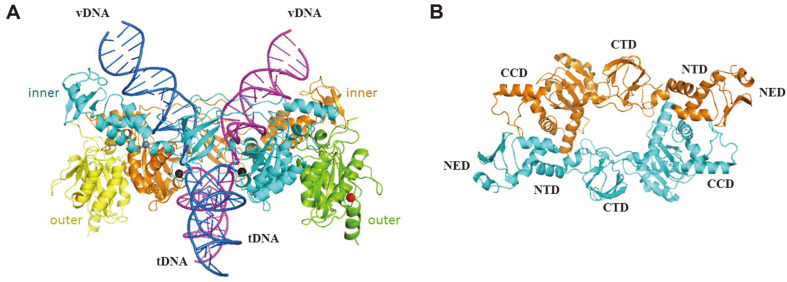
Structure of PFV IN–product complex. PDB ID 3L2R and 3OS2 were used for creating a rigid body. (**A**) Orthogonal view of tetrameric PFV IN in complex with two vDNA ends inserted into a tDNA. The two inner subunits are shown in cyan and orange, and the two outer subunits are shown in green and yellow. The two DNA molecules are shown in blue and magenta, each consisting of one vDNA end and half of the tDNA. (**B**) Ribbon diagram of two inner subunits of PFV IN intasome.

In 2010, the crystal structure of the 3'-end-processed PFV synaptic complex provided the first observation of any retroviral intasome [59]. Today, the PFV intasome can be considered a minimalist assembly with the lowest- order IN-to-vDNA stoichiometry among the retroviral intasomes already characterized [60]. The PFV intasome is a two-fold symmetric complex harboring four IN subunits, with vDNA ends synapsed between a pair of IN dimers [59](Fig. 2A). The inner IN molecules of each dimer are responsible for all interactions with vDNA and provide their active sites to catalyze the integration reactions. The inner subunits interact with each other across the synaptic interface, and an underlying theme of the intasome is the swapping of the inner NTDs to function in *trans* with the opposing CCDs. The associated CTDs engage tDNA during integration and help to rigidly bridge the halves of the intasome together [58, 61](Fig. 2B). Depending on the retroviral genus, vDNA insertion points are spaced four to six base pairs apart in the host DNA [62]. The differences in size, sequence, and charge distribution among the inner CTDs suggest that each one forms a unique intasome structure, which results in unique spacing between the vDNA insertion position in the tDNA. The outer IN subunits attach to the catalytic inner molecules via the CCD–CCD dimerization interface [37].

Solution-based measures indicated that tetrameric IN forms could be the functionally relevant multimers for the integration of alpha-retroviral [50, 63] and lentiviral DNA [46, 47, 49, 64]. Although arguments and experimental evidence for comparatively larger species were also presented [46, 65, 66], the homo-tetrameric architecture observed in the PFV intasome was expected to be a universal feature of the retroviral integration machinery [66]. However, recent intasome structures from other orthoretroviruses revealed both striking complexity and diversity. Whereas the PFV intasome is constructed from four IN molecules, the mouse mammary tumor virus and Rous sarcoma virus structures contain eight IN molecules, assembled as tetramers of dimers. As in the PFV intasome, the catalytic subunits exchange their NTDs across the synaptic interface [47, 57]. The intasome is completed by insertion of CTDs, in this case, from the flanking IN dimers [26]. Collectively, the recent structural studies cited above highlight remarkable evolutionary flexibility in the construction of the intasome. Although, in principle, a functional intasome could be composed of only two catalytic IN subunits, the smallest complex observed to date is a tetrameric PFV intasome [66]. Interestingly, the NTD extension domain, NTD, and CTD of the outer IN subunits are dispensable for catalytic reactions in vitro [32, 58, 59, 67, 68]. However, the outer IN subunits were implicated in interactions with nucleosomal DNA during integration into chromatinized targets [23].

Retroviral integration may be modeled in vitro with recombinant retroviral IN and DNA oligomers mimicking the ends of the viral genome. However, they are not ideal reagents for the study of the dynamics or structure of integration complexes when monomeric IN would obscure relevant visualization. Purified intasomes are required for dynamic single-molecule analysis or structural studies. Consequently, integration assays may test the effects of IN mutations, IN inhibitors, or other chemical additives. Mackler *et al*. proposed optimal conditions for the assembly and purification of PFV intasomes [69].

Retroviral DNA is not randomly inserted into a host chromosome. Its genus-specific biases at the level of local tDNA sequences and chromatin features have been reported [70–73]. HIV-1 and some species of lentivirus favor integration within active transcription regions [74], whereas Moloney murine leukemia virus (MLV), a gamma- retrovirus, preferentially integrates into the vicinity of transcription start sites and CpG islands [75–79]. Less distinctive patterns have been observed for FVs; PFV somewhat disfavors integration within transcription regions, regardless of local gene expression activity [80, 81]. Analyses of retroviral integration sites have revealed weak palindromic tDNA sequence consensuses at the sites of vDNA joining [71,73,82–84]. A palindromic consensus implies paired symmetry within the intasome that engages the tDNA. The crystallographic analysis of PFV revealed key features of the inner IN dimer (within the tetramer) that dictate the selection of the consensus PFV integration site (-3)KWK\*VYRB*MWM(+6) (the italics mark the target site duplication of four base pairs flanking the PFV provirus, and the backslash indicates the position of vDNA joining) [36, 58].

Inhibitors of HIV-1 that target IN recognize the retroviral intasomes rather than the free enzyme [68]. Atomic resolution structures of other retroviral intasomes are therefore required to understand the mechanisms of inhibition and drug resistance.

## Genomic Analysis of FV IN DNA

The INs from FV are composed of four functionally distinct domains joined via highly divergent flexible linkers. Functional studies of these domains have been performed using IN point mutations, deletion mutations, and domain-swapping chimeric INs [42–44,52,53,85,86]. In vitro enzyme activities of PFV IN cannot be separated into independent domains. In one study, the PFV NTD deletion (Δ1–34) mutant lost DNA strand transfer activity but had detectable 3'-end processing (13%) and disintegration (33%) activity in vitro [87]. These reduced activities of the NTD deletion mutant indicate that full catalytic activity (strand transfer, 3'-end processing, and disintegration) requires the HHCC motif but also the upstream sequences of this domain. The chimeric INs with the CCD of PFV IN or HIV IN did not have any enzymatic activities due to the differences in vDNA recognition between PFV CCD and HIV CCD [42]. Such results show that the central domain of PFV IN alone is not able to recognize vDNA without help from the NTD and CTD [42]. Sequence alignment of the CCD active site of FFV IN with HIV-1 revealed that residues D107, D164, Q165, Y191, S195, and E200 affects the in vitro catalytic reactions, viral replication, and viral infectivity [43]. The single residue mutations in the active sites of FFV IN remarkably decreased the efficiency of the three IN enzymatic activities, viral replication, and viral infectivity. Additionally, the amino acid substitution of the FFV IN C-terminal residues R307 and K340 to glutamic acid dramatically affected the enzymatic activities of the protein in vitro and noticeably reduced viral production in the infected cells [86]. Such findings indicate that mutations in the CCD of FV IN might lead to improper conformational changes in the complex of the enzyme and DNA substrate.

Viral cDNA is synthesized in the cytoplasm, and this is associated with other cellular and viral proteins to form a PIC. The PIC must be transported to the nucleus, as the retroviral integration occurs within the nucleus. Various cellular nuclear-import receptors are pivotal in translocating PIC to the nucleus. Among the cellular nuclear- import receptors, the involvement of the importin a/ß heterodimer, importin 7, and transportin 3 (TNPO3) has been extensively studied [88-90]. Some studies using a monoclonal antibody targeted to FV IN suggested that Pol and Gag proteins translocate to the nucleus [88] and facilitate integration by tethering the vDNA to the host chromosome [89]. A separate study suggested that translocation of the viral genome to the nucleus is entirely dependent on IN [90]. It has also been shown that the FV genome, as well as Gag proteins, can access the nuclei of growth-arrested cells, indicating that like lentiviruses, the FV PIC can also translocate across an intact nuclear membrane. Nevertheless, cells transduced with IN-deficient vectors reveal no Gag proteins within the nuclei, suggesting that the Gag protein alone is insufficient for PIC translocation to the nucleus and that IN is an absolute requirement in growth-arrested cells [91]. Members of the retroviral family possess nuclear localization signals at different IN residues [92]. PFV IN possesses a potent nuclear localization signal in its CTD spanning residues 289–371; an amino acid mutation study revealed that some residue positions (308, 313, 318, 324, and 329) notably affect nuclear localization [93]. TNPO3 is a karyopherin involved in transporting phosphorylated serine/ arginine-rich proteins between the cytoplasm and nucleus, and it is known to be a key factor involved in HIV-1 replication [94, 95]. In one study, TNPO3 knockdown reduced PFV production in BHK-21 cells and human 293T cells [94]. Furthermore, the interaction of TNPO3 with PFV IN was reduced, underscoring that IN–TNPO3 interactions are critical for the nuclear import of PFV PIC through the nuclear pore complex [95].

FVs are complex retroviruses that regulate their gene expression by unique features: a spliced *pol* transcript and an internal promoter directing the expression of the APOBEC-antagonizing protein Bet and the viral *trans*- activator [96–100]. FV synthesizes Pol independently of Gag. The FV Pol precursor is cleaved only once between reverse transcriptase and IN by a protease (PR), resulting in a PR/reverse transcriptase and an IN protein [97, 98, 101]. The FV genome encodes four central purine-rich elements (A, B, C, and D), which are localized within the IN-encoding region [102]. The A and B elements are essential for PR dimerization and activation, the C elements are required for Gag expression [102, 103], and the D elements act as a polypurine tract during reverse transcription [104]. Pol mutants lacking IN have defects in PR activity and Pol packaging into virions. When PR is part of Pol, Pol can form dimers because of IN–IN interactions [105]. However, recently, contradictory results on FV PR activity were published. One study suggested that IN is involved in the regulation of PR [106]. This study proposed that FV IN is required for Pol encapsidation and that PR activity is regulated by viral RNA, not IN.

## Prospective Functions of FV IN

FV INs are highly appropriate for structural studies of the retroviral synaptic complex. Recombinant PFV IN is capable of executing 3'-end processing, half-site strand transfer, and concerted integration in vitro under stringent conditions [87, 107]. In the case of PFV IN, it is capable of utilizing preprocessed oligonucleotide donor DNA as short as 16 base pairs to carry out concerted strand transfer, almost exclusively [24]. However, HIV-1 IN requires DNA molecules with several hundred base pairs and additional substances, such as dimethyl sulfoxide/ polyethylene glycol, for concerted integration [108, 109]. Moreover, PFV IN forms much stronger complexes with synthetic DNA duplexes that mimic the terminal sequences of the vDNA long terminal repeat U5 domains, much faster than DNA association with HIV IN (approximately five-fold faster) [110–113].

Structural studies of FV IN–DNA complexes have provided a breakthrough for understanding the mechanisms of retroviral integration site selection [58, 59]. Each retrovirus genus shows a unique insertion pattern, which is regulated by cellular and viral factors, as well as by the local DNA conformation and chromatin structure at the site of integration. An improved understanding of the integration site selection process has recently emerged with the advent of next-generation sequencing methods to identify insertion sites [59], the resolution of the architecture of the PFV integration machinery [114], and the cellular cofactors involved in the process [115]. Aiyer *et al*. investigated the MLV IN domain structure to identify tDNA binding residues, the CCD α2 helical region, and the CTD β1–β2 loop [114]. The results showed that the MLV IN CCD is highly assisted by the PFV intasome and that the MLV IN CTD interchanges with PFV IN CTD. These findings indicated that FV INs are promiscuous for species-specific retroviral integration site selection and IN functional studies for HIV-1 integrase strand transfer inhibitors in terms of their target commitment, higher solubility, faster kinetic properties, and a broad range of substrates compared with HIV IN [28, 44].

Vector systems based on different retroviruses are widely used to achieve stable integration and expression of transgenes due to vector-genome integration into host cell chromosomes. Transient genetic manipulation systems that are based on integration- or reverse transcription-deficient gamma-retroviral vectors bear the risk of detrimental mutagenesis, which became apparent in several clinical trials in which vector genome integration led to oncogene activation [116]. FVV systems provide a useful and novel tool for the efficient transient genetic manipulation of target tissues by the stable delivery of non-viral nucleic acids. Self-inactivating, clinically-relevant FVV systems [117-120] have been developed for gene therapy in large animal models and proven effective [121, 122]. By using IN modification or hybrid systems with other proteins, it is possible to alter the integration profile of FVs, reducing the frequency of retroviral vector integration sites [123]. Deyle *et al*. generated non-integrating FVVs by introducing point mutations into active sites of the FV IN CCD sequence and demonstrated that cells infected with non-integrating FVVs expressed transgenes and became progressively diluted in the dividing cell population [124]. FVs are the most promising vectors for large transgene delivery because they have the largest mammalian retroviral genome [117, 125, 126]. Sweeney *et al*. investigated the ability of FVVs to incorporate the simian macaque FV envelope using physiological promoters to efficiently deliver large transgene cassettes by inserting increasing lengths of the dystrophin open reading frames in an FVV before quantifying the packaged vector RNA and integrated DNA [127, 128]. Molecular assays showed that a 12-kb insert could be packaged, delivered, and integrated into a target human cell genome at a sufficient titer for ex vivo gene therapy. This approach allows greatly exceeding the maximum insert size of lentivirus vectors of approximately 7 kb, making FVV an excellent candidate for the stable and efficient transfer of large transgenes.

Besides *Spumavirus*, four other genera have now been identified in the Spumaretrovirinae subfamily (*Prosimiispumavirus*, *Bovispumavirus*, *Felispumavirus*, and *Equispumavirus*), justifying the recent increase in the number of studies on FVs. FV INs have many advantages for the study of the structural and catalytic properties of retroviral INs. FV IN has a similar sensitivity to HIV-1 inhibitors, and the crystal structure of the PFV intasome, as the only determined retroviral intasome to date, will help to investigate and develop novel HIV-1 INSTIs. This review should provide insights into the biochemical activities and structural and genomic properties of FV INs. It also presents the reasons for using FV IN as a surrogate model to study the structural basis of anti-retroviral therapy, as well as to improve the safety and efficiency of FVV systems.
